# A microbial driver of clay mineral weathering and bioavailable Fe source under low-temperature conditions

**DOI:** 10.3389/fmicb.2022.980078

**Published:** 2022-08-22

**Authors:** Jaewoo Jung, Hyun Young Chung, Youngtak Ko, Inkyeong Moon, Yeon Jee Suh, Kitae Kim

**Affiliations:** ^1^Global Ocean Research Center, Korea Institute of Ocean Science and Technology, Busan, South Korea; ^2^Korea Polar Research Institute, Incheon, South Korea; ^3^Department of Polar Science, University of Science and Technology, Incheon, South Korea; ^4^Deep-Sea Mineral Resources Research Center, Korea Institute of Ocean Science and Technology, Busan, South Korea

**Keywords:** microbe-mineral interaction, psychrophilic bacteria, biomineralization, Fe sources, illite (IMt-1)

## Abstract

Biotic and abiotic Fe(III) reduction of clay minerals (illite IMt-1) under low-temperature (0 and 4°C, pH 6) was studied to evaluate the effects of bioalteration on soil properties including clay structure and elemental composition. The extent of Fe reduction in bioreduced samples (∼3.8 % at 4°C and ∼3.1 % at 0°C) was lower than abiotic reduction (∼7.6 %) using dithionite as a strong reductant. However, variations in the illite crystallinity value of bioreduced samples (°Δ2θ = 0.580–0.625) were greater than those of abiotic reduced samples (°Δ2θ = 0.580–0.601), indicating that modification of crystal structure is unlikely to have occurred in abiotic reduction. Moreover, precipitation of secondary-phase minerals such as vivianite [Fe^2+^_3_(PO_4_)_2_^⋅^8H_2_O] and nano-sized biogenic silica were shown as evidence of reductive dissolution of Fe-bearing minerals that is observed only in a bioreduced setting. Our observation of a previously undescribed microbe–mineral interaction at low-temperature suggests a significant implication for the microbially mediated mineral alteration in Arctic permafrost, deep sea sediments, and glaciated systems resulting in the release of bioavailable Fe with an impact on low-temperature biogeochemical cycles.

## Introduction

Microbe–mineral interactions have been studied for the last three decades across many fields, including mineral diagenesis, nutrient cycling, biomineralization, organic matter maturation, and the planetology of outer space, to understand environmental processes. This is because microbes and clay minerals are ubiquitous in natural sediments and play a significant role in environmental processes, such as surface area, cation exchange capacity, and clay particle flocculation (Pollastro, 1993; Kim et al., 2004, 2012; Dong et al., 2009; Bishop et al., 2013). According to previous studies, microbial Fe(III) respiration can catalyze the alteration of Fe oxides and Fe-bearing clay minerals in mesophilic and thermophilic conditions representing the surface and deep biosphere (Kostka et al., 1999; Zhang et al., 2007a; Jaisi et al., 2011; Stucki, 2011; Koo et al., 2014; Kim et al., 2019). Mineral alteration by psychrophilic bacteria in Antarctic marine sediments (Jung et al., 2019) were recently reported, providing scientific communities, such as those conducting mineralogy and biogeochemistry, with new evidence for microbial mineral alteration in the cryosphere. Microbes have been detected in diverse glacio-marine environments (Mikucki et al., 2009), ice sheets (Karl et al., 1999), permafrost (Margesin et al., 2016), and subglacial lakes (Gill-Olivas et al., 2021), introducing growing evidence (Sharp et al., 1999; Skidmore et al., 2005; Wadham et al., 2010; Montross et al., 2013) for the possibility that microbes may play a role in biogeochemical weathering, even at low-temperatures (Montross et al., 2013; Nixon et al., 2017). Biogeochemical reactions can modify in the oxidation state of iron in mineral structure resulting in physical and chemical properties of clay minerals (Kim et al., 2004), which are generally sensitive to redox conditions in the system (Gorski et al., 2013). These physicochemical changes in bioreduced clay minerals increase the amount of residual Fe(II) in clay structure and Fe in solution form, suggesting that the Fe released from sediments could be a consequence of microbial Fe(III) reduction in the clay structure (Stucki, 2011; Koo et al., 2014). However, microbial mineral alteration at low-temperature is still poorly understood because it challenges the conventional concept of kinetic and thermodynamic models.

Illite crystallinity (IC), also known as the Kübler index, has frequently been used as a proxy for low-grade metamorphism (Cashman and Ferry, 1988; Eberl and Velde, 1989) due to it being closely linked with burial temperature and time, fluid pressure, lithology, and illite composition (Weaver et al., 1971; Frey, 1987). IC comprises the half-height width of illite 10-Å peak from XRD profiles (Kübler, 1964) that reflects X-ray scattering domain size and structural distortions (Eberl and Velde, 1989), and it measures crystal alterations (Roberts and Merriman, 1985). Recently, analysis of IC has been employed beyond metamorphism to reconstruct paleoclimate conditions such as Holocene warming (Pandarinath, 2009; Wang and Yang, 2013; Jung et al., 2019). Chemical weathering in organic-rich sediments is accelerated in wet and warm monsoonal conditions, resulting in the alteration of illite structure and a corresponding change in IC. Thus, IC has been proven to have a broader impact than simply as a diagnostic for metamorphism. However, none of these studies have taken into account specifically the biotic/abiotic effects on IC. Here, we addressed this issue by conducting low-temperature biotic/abiotic reduction experiments (0 and 4°C, pH 6) designed to simulate cold environments and compared the degree of alteration by biotic and abiotic processes.

## Materials and methods

### Material, bacterial strain, and media preparation

Illite (IMt-1) (Ca_0.01_Na_0.08_K_1.58_)(Al_2.78_Fe^3+^_0.67_Fe^2+^_0.08_ Mg_0.47_)(Si_6.89_Al_1.11_)O_20_(OH)_4_, purchased from the source clays repository of the Clay Minerals Society, was used in the present study. The IMt-1 contained 12.3 % total Fe content, and the Fe(II)/total Fe ratio was 0.10 (Bishop et al., 2011). Size fractions < 2°μm of IMt-1 were separated by gravitational settling in a deionized water column and then freeze-dried to acquire a homogeneous illite (Köster et al., 2019). Psychrophilic bacteria (*Shewanella vesiculosa* sp.), which are known to be cold-adapted and cold-tolerant facultative Fe-reducing bacteria (Bozal et al., 2009), were isolated from King Sejong Station, Antarctica, by the Korea Polar Research Institute (KOPRI). *S. vesiculosa* were grown aerobically in a Luria–Bertani broth liquid medium at 15°C for 7°days to increase cell density and activity. The cells were then washed three times with 0.1 mM NaCl in order to remove the residual medium (Myers and Nealson, 1988). The final cell density for the batch experiments was ∼1.0 × 10^7^ CFU/mL, which was determined by viable-cell count and optical density measurement at 660 nm with a UV/VIS spectrometer (DR4000UV, Hach, Loveland, CO, United States). Washed cells were inoculated in the N_2_-purged M1 medium with IMt-1 (4 g/L) as the sole electron acceptor and with Na-Lactate (20 mM) as the electron donor for the microbe–mineral interaction (Table 1). The medium was buffered by MOPS to maintain pH 6 during the reaction. Control and chemical reduction sets were prepared in the same way as the experimental set, except that the microbes were not suspended in the solution. The abiotic chemical reduction set (2 h) was reduced using dithionite (Na_2_S_2_O_4_) as a strong reductant. A sample of 100 mL of this suspension was placed in each serum bottle (4 and 0°C). The reaction was stopped at time points of 1, 2, 4, 8, 12, and 24°weeks by freezing the samples in a deep freezer (−70°C) and then thawing them in ambient temperature water for further analysis.

**TABLE 1:** Experimental settings for bioreduced, control, and chemical reduced condition.

Experimental set	Composition		
	Clay mineral	Medium	Dithionite
Bioreduced	v	v	
Control	v	v	
Chemical reduced		v	v

pH	6.0		
Temperature	0°C, 4°C		
Medium composition	M1 Basal salts, M1 Trace element, M1 Phosphate, M1 metal supplement, MOPS, 15 mM Sodium selenate, 0.2 M NaHCO_3_, M1 Amino acid, 2 M C_3_H_5_O_3_Na, 2 M HCOONa		

### Chemical analysis

The structural Fe of IMt-1 was extracted by 3.6 N H_2_SO_4_ and 48 % HF, and then 1,10-phenanthroline reagent was added to the samples (Stucki, 1981). Triplicate copies of each set were prepared to measure the Fe(II)/Fe_total_ and minimize the measuring error. The concentration of the Fe(II) was measured with a UV/VIS spectrometer (DR4000UV, Hach, Loveland, CO, United States) at 510 nm of wavelength. Hydroxylamine was added to reduce residual Fe(III) to Fe(II), and then Fe(II) concentration was measured again for Fe_total_ content. The concentration of dissolved Fe [Fe(II)_aq_ + Fe(III)_aq_] in the solution was determined by inductively coupled plasma atomic emission spectroscopy (ICP-AES; ICAP 7000, Thermo Scientific, Germany) at the Korea Polar Research Institute (KOPRI). The liquid aliquots were collected after centrifugation (11,000 rpm, 3 min) and then filtered with a 0.45°μm filter to remove residual particles (Kim et al., 2010; Jeong et al., 2012).

### X-ray diffractometer

X-ray diffraction (XRD) analyses were obtained at 30°kV and 10°mA with an X’PERT-PRO automated diffractometer utilizing Cu-Kα radiation. XRD profiles were recorded at a scan speed of 0.02 step and 1.0°/min over the range of 2θ angles (2–40°2θ), and then Crystallographica Search-Match software (version 2.0.3.1) was used to identify the mineralogical change. IC refers to the full width at half-maximum height (FWHM) of illite (001) XRD peak (Kübler, 1964; Jaboyedoff et al., 2001) utilizing OriginPro8 software after the background removal by Chebyshev polynomial (≤ 20 coefficients) and the pseudo-Voigt function suggested by Thompson (Thompson et al., 1987).

### Electron microscopic observation

A field emission scanning electron microscope (FE-SEM; Gemini500, Zeiss, Germany) with energy dispersive X-ray spectroscopy (Ultim max100, Oxford Instruments, United Kingdom), operating at a working distance of 10 mm and 15°keV, was used to observe the precipitation of secondary-phase minerals. SEM specimens were prepared following a method to improve their image resolution and elemental composition (Dong et al., 2003a). The prepared specimens were air-dried for 24 h prior to Pt coating 10 nm in thickness.

## Results

### Biotic/abiotic Fe reduction

**FIGURE 1:**
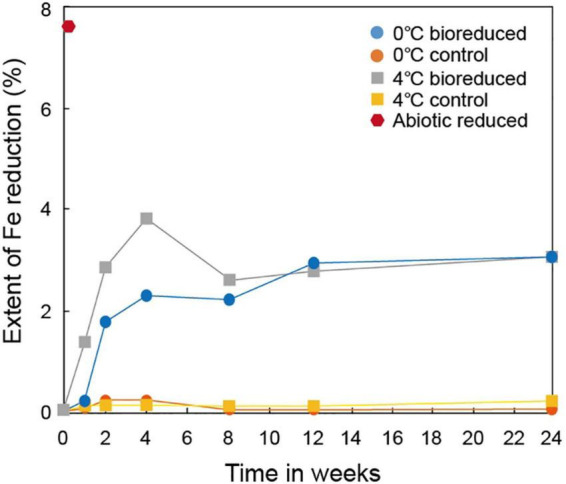
Extent of biotic and abiotic Fe reduction in IMt-1 during batch experiment at various temperatures (0 and 4°C) and incubation times.

A series of dissolution experiments (0 and 4°C at pH 6) was performed using IMt-1 with *S. vesiculosa* to estimate Fe(II) generated biotically and abiotically (Figure 1). The extent of Fe(III) reduction in the bioreduced sample at 0°C reached ∼2.3% after 4°weeks and then slightly increased to ∼3.1% after 24°weeks of incubation. The bioreduced sample at 4°C reached up to ∼3.8% after 4°weeks and then decreased to ∼2.6% after 8°weeks of incubation. After that, the extent of Fe(III) reduction slowly increased to ∼3.1% after 24°weeks. After the first 4°weeks of incubation, the highest rate of Fe(III) reduction was observed at both temperatures, while no measurable Fe(III) reduction (∼0.2%) was shown in the control samples (0 and 4°C). Abiotic reduction using the dithionite reduced more Fe(III) in IMt-1 (∼7.6%) than biotic reduction.

**FIGURE 2:**
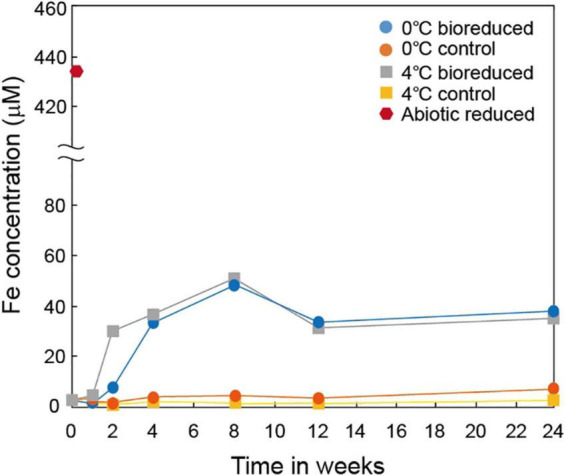
Production of total dissolved Fe from IMt-1 during batch experiment at various temperatures (0 and 4°C) and incubation times.

The concentration of the total dissolved Fe upon biological dissolution from IMt-1 at both temperatures (Figure 2) increased rapidly for up to 8°weeks of incubation (44.2 μM in 0°C and 47.3 μM in 4°C) and decreased after 12°weeks (32.9 μM in 0°C and 26.8 μM in 4°C), and 24 weeks (36.5 μM in 0°C and 32.1 μM in 4°C). Abiotic reduction showed the highest concentration of dissolved Fe (431.5 μM). On the other hand, a small variation in dissolved Fe (6.0 μM in 0°C and 1.3 μM in 4°C) in the solution was measured in the control samples.

### Mineralogical change

**FIGURE 3:**
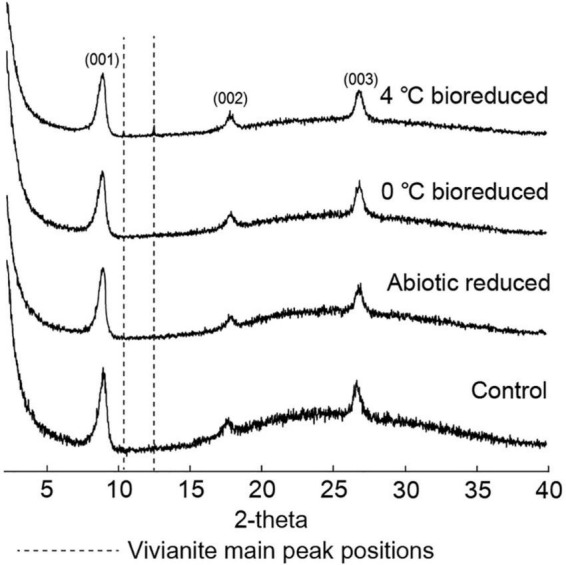
X-ray diffraction (XRD) profiles for illite and vivianite main peaks for bioreduced, abiotic reduced, and control samples after 24°weeks.

The XRD profiles (Figure 3) of the IMt-1 samples at pH 6 for the 24°weeks of incubation all showed main peaks of illite, including (001), (002), and (003) peaks. The values of IC in °Δ2θ showed variation with conditions in a range of 0.580–0.613 (0°C bioreduced IMt-1), 0.580–0.625 (4°C bioreduced IMt-1), and 0.580–0.601 (abiotic reduced IMt-1). A particularly abrupt increase in corresponding IC values for bioreduced IMt-1 was noticed (Table 2); however, a small variation in IC values (0.580–0.583 °Δ2θ) was measured in the control samples (0 and 4°C).

**TABLE 2:** Full width at half maximum (°Δ2θ) of biotic/abiotic reduced IMt-1.

Time	0°C bioreduced	0°C control	4° bioreduced	4°C control	Abiotic reduced
Time zero	0.580	0.580	0.580	0.580	0.580
2 h					0.601
1 week	0.582	0.580	0.583	0.581	
2 weeks	0.587	0.582	0.599	0.582	
4 weeks	0.608	0.582	0.621	0.581	
8 weeks	0.611	0.583	0.623	0.582	
12 weeks	0.613	0.582	0.624	0.584	
24 weeks	0.613	0.583	0.625	0.583	

**FIGURE 4:**
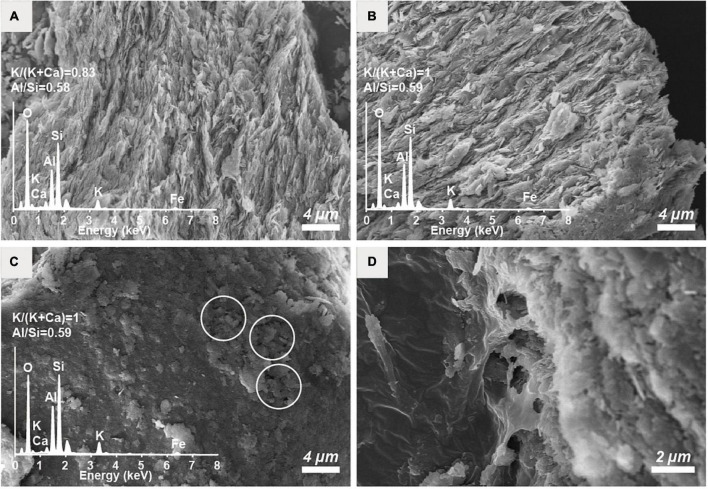
SEM images of the **(A)** unaltered initial IMt-1, **(B)** chemically reduced IMt-1, **(C)** bioreduced IMt-1 after 24°weeks incubation, and **(D)** magnification of dissolution pits in bioreduced IMt-1.

**FIGURE 5:**
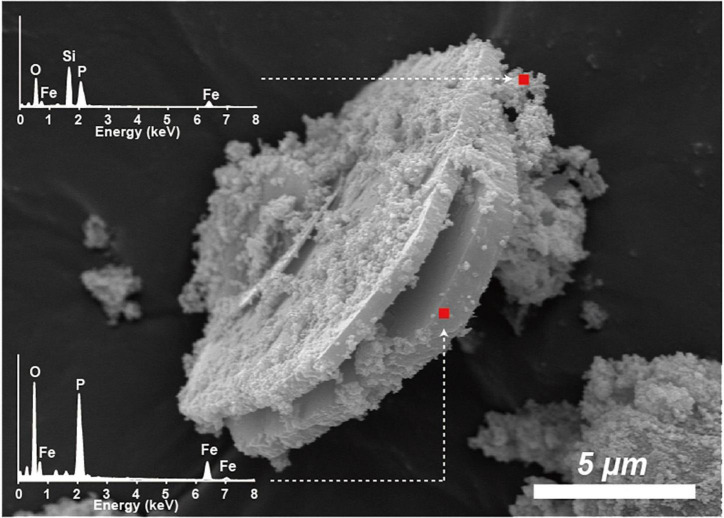
SEM image of euhedral vivianite crystal and nano-sized aggregated silica in bioreduced sample after 24°weeks incubation. Elemental composition of Fe, P, and O in EDS measurement.

The unaltered initial IMt-1 showed a bulky-, flaky-looking texture (Figure 4A), and corresponding elemental compositions of Al/Si (0.58) and K [K/(K+2Ca) = 0.83] in SEM-EDS were observed. In the abiotic reduced IMt-1, initial illite remained at the end of the incubation period, but the elemental compositions of Al/Si (0.59) and K [K/(K+2Ca) = 1] were increased (Figure 4B), and no interlayer Ca was measured. After bioreduction, bioreduced IMt-1 displayed altered crystalline boundaries and small euhedral crystals of illite (Figure 4C). The elemental compositions of Al/Si (0.59), K content [K/(K+2Ca) = 1], and no interlayer Ca were measured. Many dissolution pits were observed in the bioreduced samples (Figure 4C). The magnified image of bioreduced IMt-1 (Figure 4D) showed the size of dissolution pits (∼1–2°μm). A new mineral phase, vivianite [Fe^2+^_3_(PO_4_)_2_⋅8H_2_O], was detected at 11.15 °2θ and 13.15 °2θ in the XRD profile only for the 4°C bioreduced sample after 24°weeks of incubation, in contrast to the abiotic reduced and control samples (Figure 3). Direct observation of euhedral crystal at approximately 10–20°μm in size and elemental composition of Fe, P, and O in EDS measurement indicated the precipitation of secondary-phase mineral precipitation (Figure 5). Moreover, nano-sized and aggregated biogenic silica was detected only in the bioreduced sample.

## Discussion

### Biotic/abiotic reduction of clay minerals

The extent of microbial Fe(III) reduction in clay minerals varies depending on elements of the experimental setting, such as the type of bacterial species, concentration of inoculated cells and minerals, medium chemistry (pH, buffer, and elemental composition), temperature, and presence of electron shuttle (Kostka et al., 1999; Jaisi et al., 2005, 2007; Furukawa and O’Reilly, 2007; Zhang et al., 2007a,b). Moreover, the limited reduction of structural Fe(III) in clay minerals has been attributed to multiple factors (Roden and Wetzel, 2002; Jaisi et al., 2007). Additional inoculation of fresh cells is the most effective way for electron acceptors such as clay minerals or Fe-oxides to promote the biotic reaction (Urrutia et al., 1998). However, none of these studies have compared the degree of biotic/abiotic effects on mineral alteration, such as the modification of IC. Microbial alterations of IMt-1 responded to increases in the extent of Fe(III) reduction (Figure 1), and consequently, the soluble form of Fe concentration (Figure 2) from reductive dissolution was clearly displayed in the modification of mineral structure (Table 2) and precipitation of secondary phase minerals (Figures 3, 5). Interestingly, variations in IC value abruptly increased in bioreduced samples compared with abiotic reduced samples (Table 2), which showed a much higher extent of Fe(III) reduction. In other words, low crystalline illite (high IC value) has a correlation with the activity of microbial Fe respiration, which causes the alteration of illite structure in anaerobic low-temperature conditions. Progressive changes in morphology of bioreduced IMt-1 (Figure 4C), compared with abiotic reduced and control samples (Figures 4A,B), supported a reductive dissolution of clay minerals and secondary-phase mineral formation (Figure 5), as shown in the solution chemistry (Figure 2) and mineralogy (Figure 3 and Table 2). Furthermore, many dissolution pits in bioreduced IMt-1 showed similar sizes to microbes (Figure 4C). The magnified image of bioreduced IMt-1 (Figure 4D) further confirmed that the size of the dissolution pits was similar to those of microbes (∼1–2°μm). The formation of nano-sized aggregated biogenic silica also strongly supported the dissolution of illite (Figure 5). Furthermore, biogenic minerals show much smaller in crystal size (Zhang et al., 2007a) and have fewer impurities than those that are inorganically formed (Carvallo et al., 2008).

### Mineral transformation by microbial reduction

**FIGURE 6:**
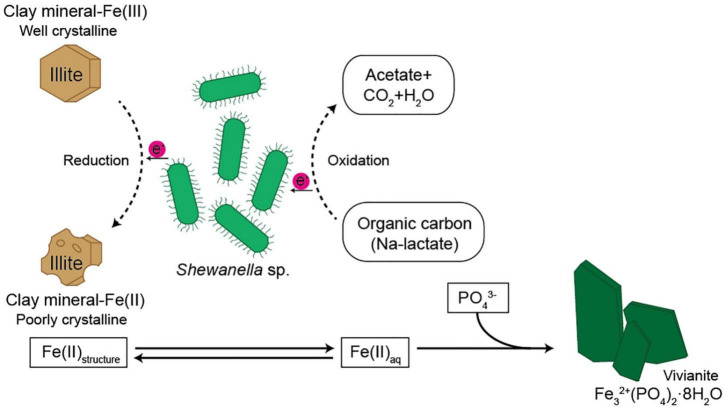
Schematic diagram showing possible pathway of Fe cycle and electron transfer from microbe to the structural Fe(III) of the clay minerals.

Diverse microorganisms have been shown to get energy from Fe respiration using solid minerals to maintain their growth and metabolism (Kostka et al., 1996, 1999; Lovley, 2004), resulting in mineralogical and chemical changes. Generally, two possible mechanisms for mineral transformation by microbial Fe(III) reduction have been proposed: (1) solid-state reaction and (2) dissolution–precipitation (Dong et al., 2009). Microbial Fe(III) reduction in clay minerals occurs without any dissolution in the solid state model. If any, mineralogical modifications such as dissolution are very small, and reaction is completely reversible after reoxidation (Gates et al., 1996; Kashefi et al., 2008). However, mineral dissolution occurs, either before or after microbial Fe(III) reduction, in the dissolution–precipitation model. In this case, reactions are irreversible, and secondary-phase minerals are often observed resulting from bioreduction (Dong et al., 2003a; Jaisi et al., 2007; Zhang et al., 2012). The euhedral vivianite crystals and nano-sized aggregated biogenic silica observed in this study (Figure 5) demonstrated that psychrophilic bacteria were capable of dissolving illite IMt-1 and precipitating new minerals in low-temperature conditions (Figures 5, 6). The initial increase in concentration of the total dissolved Fe in bioreduced samples (Figure 3) may result from the reductive dissolution of IMt-1, and the subsequent decrease could be attributed to the precipitation of vivianite (hydrated iron phosphate mineral). The dissolution of IMt-1 and formation of biogenic secondary-phase minerals suggest that psychrophile-mediated redox cycling of IMt-1 is not a reversible reaction. Moreover, the bioreduced IMt-1 was more enriched in the interlayer cation (K) than abiotic-reduced sample (Figure 4C). These additional cations were expected to rebalance the negative charge resulting from the Fe-reduction [Fe(III) to Fe(II)] in the octahedral sheet of the illite structure (Zhang et al., 2012). Over an extended time in natural soil environments, Fe-bearing minerals such as smectite, illite, smectite–illite (S-I) mixed-layer, and Fe-oxides may undergo reductive dissolution in anoxic conditions and supply the bioavailable iron (Stucki, 1988; Kostka et al., 1999; Stucki and Kostka, 2006; Jaisi et al., 2011; Liu et al., 2012). On the other hand, they could have been re-oxidized partially when they were exposed to oxygen and other oxidants (Stucki, 2011). Therefore, Fe-bearing clay minerals may go through repetitive cycles of the Fe redox state and play an important role in the elemental cycle in low-temperature environments.

### Environmental implications

The microbe–mineral interactions are commonly anaerobic reactions and are found in various soils and sediments distributed in glacial, deep sea, and hydrothermal vents (Bishop et al., 2013). A new challenge for testing the modification of IC for the biogeochemical reaction was initiated to simulate cold environments and compare the degree of alteration by biotic and abiotic processes. It is the first measured evidence of IC responding to biotic and abiotic Fe reduction at low-temperature. These results also can be applied to search for biosignatures in clay minerals on the primitive Earth and Mars (Bishop et al., 2013). Furthermore, bioavailable iron is a necessary nutrient for the growth and metabolic process of phytoplankton in marine environments (Morel and Price, 2003; Wadley et al., 2014). For this reason, the increase in primary productivity is restricted due to insufficient iron in some areas of the global ocean that causes outgassing of upwelled CO_2_ that serves as a regulator of climate change (Martin, 1990). However, the source of bioavailable iron to the global ocean still remains an open question. Low crystalline illite (high value of IC) has a strong relation with the activity of microbial Fe respiration, which causes the alteration of illite structure in low-temperature anaerobic conditions (Jung et al., 2019). The psychrophilic Fe-reduction in illite resulted in structural and chemical modification similar to the consequences presented in our previous reports for mesophilic (Dong et al., 2003b; Kim et al., 2004) and thermophilic reactions (Zhang et al., 2007a). Illite appears prominently on continental shelves (Petschick et al., 1996; Wang and Yang, 2013), and the concentration of dissolved Fe(II) is higher near the coastline than in the open ocean (Schlosser et al., 2012), suggesting that bioavailable iron may be transported from terrigenous sediments to the ocean through microbial Fe(III) reduction in Fe-rich sediments. An approximation of the total Fe released to the solution through microbial Fe(III) reduction in Fe-rich smectite was about ∼5% of calculated total structural Fe in our previous experiment for psychrophilic microbe and NAu-2 interaction (Keeling et al., 2000; Jung et al., 2019). In consideration of the clay content in marine sediments [clay content is generally < 30% and partly < 10% of the bulk sediment (Petschick et al., 1996)], microbial activity positively related to the modification of illite structure corresponding to redox conditions plays an important role in nourishing the bioavailable Fe(II) in global oceans.

## Conclusion

The evidence of microbially weathered illite was identified through the progressive increase in extent of Fe reduction and dissolved Fe concentration, and dissolution features corresponding to the increases in K-content (K/K+Ca) and Al/Si. The extent of Fe reduction in bioreduced samples was lower than abiotic reduction using dithionite as a strong reductant. However, variations in the illite crystallinity value of bioreduced samples were greater than those of abiotic reduced samples, suggesting that modification of mineral structure is unlikely to have occurred in abiotic reduction. Moreover, precipitation of secondary-phase minerals such as vivianite and nano-sized silica were shown as evidence of reductive dissolution of Fe-bearing minerals that is observed only in a bioreduced setting. In summary, our observation of a previously undescribed microbe–mineral interaction at low-temperature suggests an important implication for the microbially mediated mineral alteration in Arctic permafrost, deep sea sediments, and glaciated systems resulting in the supply of bioavailable Fe with an impact on biogeochemical cycles in low-temperature environments.

## Data availability statement

The original contributions presented in this study are included in the article/supplementary material, further inquiries can be directed to the corresponding author.

## Author contributions

JJ designed the overall research concept, data interpretation, and drafted the manuscript. HC produced data, including ICP and SEM data with the help of YS, and IM developed the concept of pathway of Fe cycle. YK and KK interpreted overall data and revised the manuscript. All authors have contributed to the writing of the manuscript, read, and agreed to the published version of the manuscript.
